# Mobile Apps for Oncology Health Care Professionals: Mapping and Assessment Study

**DOI:** 10.2196/71203

**Published:** 2026-02-11

**Authors:** David Liñares, Iolie Nicolaidou, Andreas Charalambous, Daniela Cabutto, Deborah Moreno-Alonso, Clara Madrid Alejos, Norbert Couespel, Noemí López-Rey, María José Fernández-Domínguez, Carme Carrion, Ana Clavería

**Affiliations:** 1 Área Sanitaria de Vigo I-Saúde Group, Instituto de Investigación Sanitaria Galicia Sur, Servizo Galego de Saúde, Centro de Salud de Rosalía de Castro Vigo Spain; 2 Galician Agency for Health Technology Assessment, Avalia-t, Galician Agency for Health Knowledge Management Santiago de Compostela, Galicia Spain; 3 Research Network in Chronicity, Primary Care and Health Promotion (Red de Investigación en Cronicidad, Atención Primaria y Promoción de la Salud) Barcelona, Catalonia Spain; 4 Nursing Department Cyprus University of Technology Limassol Cyprus; 5 University of Turku Turku Finland; 6 eHealth Lab Research Group School of Health Science Universitat Oberta de Catalunya Barcelona Spain; 7 Àrea suport integran en càncer, Institut del Càncer i Malalties de la Sang Hospital Clínic de Barcelona Barcelona, Catalonia Spain; 8 e-Oncologia and Cancer Education Research Unit Institut Català d'Oncologia Barcelona Spain; 9 European CanCer Organisation Brussels Belgium; 10 School of Medicine Universitat de Girona Girona, Catalonia Spain

**Keywords:** mobile apps, neoplasms, technology assessment, biomedical, telemedicine, quality of health care

## Abstract

**Background:**

The use of mobile apps in oncology has been expanding rapidly, encompassing prevention, treatment, and patient support. These technologies hold significant potential to improve care delivery and enhance the efficiency of health care services. However, their integration into clinical practice faces important challenges. A key issue lies in the difficulties health care professionals (HCPs) encounter when selecting apps that adequately meet their specific needs and comply with appropriate standards of quality and clinical effectiveness. This lack of robust evidence on the availability, adoption, and evaluation of mobile apps designed for cancer care professionals not only hinders their wider adoption but also restricts their potential to serve as reliable tools in oncology practice.

**Objective:**

This study aims to map the landscape of free mobile apps for cancer prevention, treatment, therapy, or support for HCPs, and assess the quality of the apps identified.

**Methods:**

A systematic search was conducted on Google Play and Apple App Store in June 2023 and December 2024 using predefined oncology- and professional-related keywords, following PRISMA (Preferred Reporting Items for Systematic Reviews and Meta-Analyses) guidelines. Two independent reviewers (DL and AC) assessed the selected apps using the Mobile App Rating Scale (MARS), which evaluates engagement, functionality, aesthetics, information quality, and subjective quality on a 5-point Likert scale. Discrepancies in ratings were resolved by a third reviewer. Descriptive statistics summarized the app quality and characteristics.

**Results:**

Out of 221 apps initially identified, 20 met the inclusion criteria and were evaluated. Most apps (15/20, 75%) supported both Android and iOS platforms, with 90% (18/20) commercially developed. The mean overall MARS score was 3.51 (SD 0.54), indicating moderate quality but with room for improvement. Only 2 apps, ONCOassist (Portable Medical Technology Ltd.) (mean 4.25, SD 0.26) and Oncology Board Review (mean 4.03, SD 0.39), surpassed the threshold of 4.0, considered good quality. ONCOassist stood out for its comprehensive functionality and high information quality, offering clinical decision support tools such as treatment protocols, prognostic calculators, and toxicity grading aligned with professional oncology practice. Prevention and support apps generally scored lower, particularly in engagement and interactive features. No app achieved a high score across all MARS domains.

**Conclusions:**

The study highlights a fragmented landscape of free mobile apps for cancer care professionals, with predominantly low to moderate quality and limited evidence to support clinical effectiveness. ONCOassist emerges as a promising tool warranting further investigation. This underscores the urgent need for standardized evaluation frameworks, regulatory oversight, and sustainable development strategies to ensure the creation and adoption of reliable, evidence-based digital health tools in oncology.

## Introduction

Cancer is the second leading cause of premature mortality and morbidity in Europe, with 4.7 million new cases and 2.1 million deaths annually in the European Union [1]. The European Cancer Control Plan reflects this political priority, further emphasized by the inclusion of cancer as one of the 5 missions in the Horizon Europe program [2,3].

In recent years, partly accelerated by the COVID-19 pandemic, telehealth apps have grown rapidly, including mobile health (mHealth) apps designed for oncology [4,5]. These apps leverage the convenience of smartphones to support cancer prevention and diagnosis, symptom monitoring, and communication between health care providers and patients [4,6]. Although available to diverse audiences, nearly 45% are designed for health care professionals (HCPs), making them the most prominent target group [7]. Their primary functions are educational, with secondary roles in supporting clinical decision-making and patient care [7]. Mobile apps may be used as stand-alone interventions or in combination with additional support material as part of broader interventions [8].

There is growing evidence that digital health technologies, such as mobile apps, can support self-management, reduce symptom burden, improve cancer care management, and enhance quality of life for patients with cancer [4,9,10]. These apps, used on smartphones or tablets, may facilitate medication adherence, symptom tracking, and disease management [11], as well as promote healthy lifestyles and access to information [6]. Despite this potential, and although HCPs generally express positive attitudes toward their use [12], actual adoption in cancer care remains limited. Evidence from the United States and several European countries indicates a mismatch between the rapid proliferation of cancer-related apps and their real-world use among both professionals and patients [5].

The adoption of mHealth apps by HCPs is hindered by several barriers. First, HCPs face difficulties in identifying appropriate apps for their needs and in evaluating the extent to which scientific evidence supports their use [9]. Moreover, no tools are currently available to help cancer patients or professionals search for high-quality cancer apps [13], making it difficult for both digital laypersons and HCPs to identify reliable options [14]. The quality of existing apps for HCPs also varies and is generally low: systematic searches have found that almost half of the tested apps were rated as deficient or insufficient [15]. Another limitation is that most cancer care apps are not developed with the involvement of health providers, which complicates their implementation [13]. Finally, cost represents an additional barrier, as 77% of health app users rely exclusively on free apps [16,17]. Consequently, the most recent evaluations of health apps have focused only on those that are freely available [18].

Given these challenges, app evaluations can help clarify the landscape. There are several approaches to evaluating apps, including content analyses, usability testing, observational studies, and efficacy trials [19]. Among these, questionnaires and scales are the most common for assessing usability in eHealth apps, although they do not always identify the specific issues that need to be addressed [20]. However, despite the increasing number of cancer-related apps, evidence about the availability and quality of free apps specifically designed for HCPs remains scarce.

To address this gap, this cross-sectional study aims to map the landscape of free mobile apps for cancer prevention, treatment, therapy, or support for HCPs, and assess the quality of the apps identified. This analysis focuses on the characteristics and overall quality of these apps, thereby contributing to the evidence base and informing future development.

## Methods

### Study Design

This study was designed to evaluate the availability and quality of apps developed for the prevention, treatment, therapy, or support of cancer for HCP during the years 2023 and 2024. The decision to conduct the study over this period was informed by the dynamic nature of mHealth technologies, characterized by rapid advancements and frequent updates.

To achieve this, a systematic search was conducted across major app download platforms, followed by a peer evaluation of each identified app. It is part of the Digital Transition and Digital resilience in Oncology (TRANSiTION) project [21], which aims to develop advanced training to equip both clinical and non-clinical professionals (ie, health managers) with essential digital skills.

### Search Strategy

A systematic search of mHealth apps related to cancer prevention, treatment, therapy, and support for HCPs was conducted in Google Play and the App Store. A completed PRISMA (Preferred Reporting Items for Systematic Reviews and Meta-Analyses) 2020 checklist is provided in Multimedia Appendix 1. For the purpose of this study, a cancer HCP is defined as any clinical professional (eg, oncologist and radiation therapist) or non-clinical professional (eg, manager and administrative staff) involved in cancer care.

The search strategy was based on methods used in previous studies [22,23]. The search was conducted using a Spain and Cyprus IP addresses. The initial search was performed in June 2023, with a follow-up search in December 2024, to compare the evolution of apps over 1.5 years. The search terms used were “oncology or cancer prevention” or “cancer treatment or cancer therapy” or “cancer support”; “health care professional*” or “doctor*” or “nurse*”, “nursing*”; “health manager*”. The terms and parameters used were identical to those used in the systematic review conducted by Cloconi et al [24], which aimed to review the digital health technologies used by HCPs and health managers in the academic literature on cancer care.

The inclusion and exclusion criteria used for the selection of apps are listed in Textbox 1.

**Inclusion and exclusion criteria for app selection.**

**Inclusion criteria**

- Apps targeted at health care professionals providing cancer prevention, treatment, therapy, or support.
- Apps available on Google Play and the Apple App Store currently.

**Exclusion criteria**

- Apps that were not available in English.
- Apps that were not freely available.
- Apps with nonfunctional links.
- Apps not related to oncology.
- Apps addressing the needs of patients, but not of health care professionals.
- Apps with nonfunctional links. Apps not related to oncology.

To be included in the data extraction, apps had to meet all inclusion criteria and none of the exclusion criteria. Google Play apps were searched using a laptop (Windows 11) by systematically entering predefined keywords. The same process was applied for the App Store, through a desktop computer (macOS 13). This approach ensured the selection of apps that are directly accessible and functional for end users.

### Data Extraction

Two independent researchers (DL and AC) collected data from descriptions of online apps (including app features and narrative text) available in the App Store and Google Play Store. The variables collected for each app included the following: app name and target group, category, objective, rating (out of 5), number of downloads, development company, cost, creation date, last update, availability, and whether an official source was cited. The technical aspects of the apps were also documented, including features such as the ability to share on social media, the requirement to log in, and the need for web access to function. Additionally, information was collected regarding potential commercial interests, including the presence of advertisements or options for purchasing services. The purposes of the apps were classified using an open-ended question that provided a brief description of the digital tool.

### App Quality Evaluation

To assess app quality, the Mobile Apps Rating Scale (MARS) was used [25]. MARS is a tool specifically designed to evaluate the quality and content of mHealth apps. MARS consists of a total of 23 items covering five dimensions: (1) engagement (5 items: fun, interest, individual adaptability, interactivity, and target group), (2) functionality (5 items: performance, usability, navigation, and gestural design), (3) aesthetics (3 items: layout, graphics, and visual appeal), (4) information (7 items: accuracy of app, description, goals, quality of information, quantity of information, quality of visual information, credibility, and evidence base), and (5) subjective quality (4 items: recommendation, times of use, pay-per-use, and overall rating). Items are assessed on a 5-point Likert scale. Items assessing information quality can also be rated as not applicable (eg, in case of missing evidence or missing visual information). Each dimension's score is the average of its items. Therefore, the score for each dimension can range from 1 to 5 (1=inadequate, 2=poor, 3=acceptable, 4=good, and 5=excellent). MARS has excellent psychometric properties [26]. In this study, all the dimensions showed adequate internal consistency (Cronbach α=0.78-0.91).

The app evaluation procedure used was consistent with that of previous studies [27]. Apps were reviewed by 2 trained reviewers (DL and AC), selected randomly from a group of experts engaged in the European project TRANSiTION on e-skills training for cancer HCPs, as mentioned previously. The reviewer team consisted of professionals trained in family medicine, psychology, and nursing, with additional specialization in health technology assessment and digital education. Before conducting the MARS evaluation, reviewers piloted a review of one app and discussed each MARS item to ensure an appropriate level of interrater consensus ratings in a hybrid session. In case the concordance between the reviewers was not reached (ie, a difference greater than one point in A, B, C, or D MARS dimensions), a third reviewer assessed the app. The lead researcher, an expert in digital health with extensive expertise in biotechnology, oversaw the MARS qualification process.

A descriptive analysis was developed, with continuous variables presented as means. Interrater reliability was examined using the intraclass correlation coefficient (ICC), calculated with a 2-way random-effects model and absolute agreement definition. The data generated were analyzed using IBM SPSS Statistics (version 25.0; IBM Corp).

### Ethical Considerations

This study did not involve human participants or the collection of personal data. It was reviewed by the Pontevedra-Vigo-Ourense Research Ethics Committee (ref: 2023/309), which indicated that no formal evaluation was required.

## Results

### Description

Within the scope of this study, searches were conducted in June 2023 and December 2024. In the initial search, 221 apps were screened, of which 18 met the inclusion criteria. Among these 18 apps, 16 underwent version updates in 2024, with the exceptions being the European Society for Medical Oncology (ESMO) Interactive Guidelines and TNM Cancer Staging Manual.

**Figure 1:**
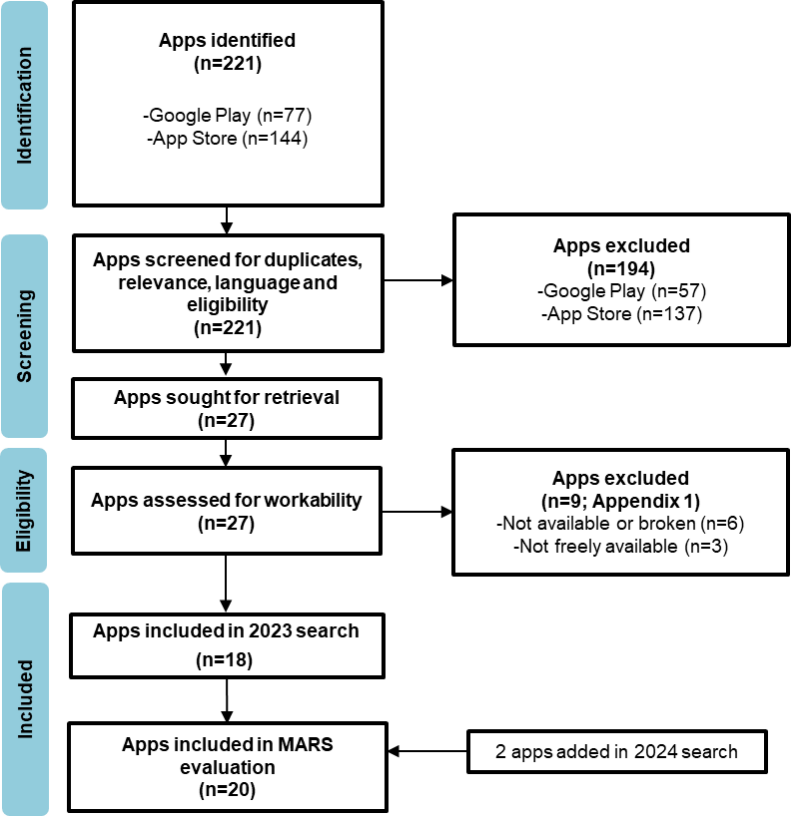
PRISMA flowchart of the identification, screening, and selection of free oncology mobile applications apps for health care professionals, based on searches conducted in Google Play and Apple App Store in June 2023 and December 2024.

The subsequent search in December 2024 identified 20 eligible apps, 18 of which had been initially detected in the June 2023 search and remained available. Consequently, this search marked the introduction of 2 new apps that were unavailable in the preceding year (Figure 1). The list of excluded apps can be found in Multimedia Appendix 2.

Of the total, 75% (15/20) of the apps are available for Android and iOS. All apps had commercial affiliations, except for 2 (ESMO Interactive Guidelines and “NCCN Guidelines” [National Comprehensive Cancer Network]), which are affiliated with non-governmental organizations. Only 45% (9/20) of the apps were created after the COVID-19 pandemic.

The apps, while related to cancer, exhibited considerable heterogeneity with respect to their focus. Several were focused on teaching users about the treatment of the disease, while others intended to prepare users for an examination. Some cancer guidelines apps were also included. Other apps even extended beyond cancer to address a range of physical or mental illnesses. None of these apps has conclusive evidence of their validity and reliability for their purpose. The final list of analyzed apps is shown in Table 1, where it is specified the app’s name, cancer type, platform, and year of creation are specified.

**Table 1:** Oncology mobile apps for health care professionals were included for evaluation in this study.

Apps	Developer	Focus: cancer type	Platform	Created
All Diseases Treatments	My Apps Studio	Learning disease treatment. Includes most types of cancer	Android	2021
All Diseases Treatments 2023	Soft Solutions	Learning disease treatment. Includes most types of cancer	Android	2022
Cervical Cancer Guide	Apps For Everyone	Cervical Cancer Guide: symptoms, causes, prevention, and treatment	Android	2024
Colon Cancer Guide	Apps For Everyone	Colon Cancer Guide: symptoms, causes, prevention, and treatment	Android	2024
Current Medical Diagnosis and Treatment	Skyscape Medpresso Inc	Diagnosis and treatment information. Includes most types of cancer	Android and iOS	2021
ESMO Interactive Guidelines	European Society for Medical Oncology	Cancer diagnosis and treatment recommendations guidelines. Includes most types of cancer	Android and iOS	2015
Fitzpatrick’s Color Atlas	Skyscape Medpresso Inc	Guideline update for dermatological disease. Includes precancerous lesions and cutaneous carcinomas	Android and iOS	2021
Harrison’s Manual of Medicine	Skyscape Medpresso Inc	Guideline update in different pathologies. Includes most types of cancer	Android and iOS	2018
Hematology & Oncology Consult	Skyscape Medpresso Inc	Hematology and oncology information. Includes most types of cancer	Android and iOS	2017
NCCN Guidelines	National Comprehensive Cancer Network	Guideline update in oncology. Includes most types of cancer	Android and iOS	2013
ONCOassist	Portable Medical Technology Ltd	Decision support by providing a range of tools (eg, treatment protocols and prognostic scores). Includes most types of cancer	Android and iOS	2015
Oncology Board Review	Higher Learning Technologies Inc	Oncology exam training. Includes most types of cancer	Android and iOS	2017
Oncology Nursing Drug Handbook	Skyscape Medpresso Inc	Cancer Treatment and drug therapy, symptom management cancer. Includes most types of cancer	Android and iOS	2018
Pediatric Disease & Treatment	Patrikat Softech	Information on children's diseases, pediatric treatments, and medical calculators. Includes bone, eye, and hematological cancers	Android	2018
Pharmacology for Nursing 2023	Pass Your Exam	Oncology exam training. Includes most types of cancer	Android and iOS	2022
Radiation Oncology Exam Review	Higher Learning Technologies Inc	Radiation exam training. Includes most types of cancer	Android and iOS	2018
Radiation Oncology Q&A Review	Skyscape Medpresso Inc	Learning radiation oncology. Includes most types of cancer	Android and iOS	2021
TNM Cancer Staging Manual	Skyscape Medpresso Inc	Classification and staging of cancer. Includes most types of cancer	Android and iOS	2021
Washington Manual of Oncology	Skyscape Medpresso Inc	Guideline update in oncology. Includes for most types of cancer	Android and iOS	2017
Williams Manual of Hematology	Skyscape Medpresso Inc	Guideline update in hematology. Includes for most types of cancer	Android and iOS	2018

Interrater reliability indicated a moderate overall level of agreement (ICC=0.66). Reliability varied across MARS dimensions, ranging from excellent for engagement (ICC=0.90) to good for functionality (ICC=0.75), moderate for aesthetics (ICC=0.64) and information (ICC=0.52), and poor for subjective quality (ICC=0.38).

The combined results of the objective assessments (ie, engagement, functionality, aesthetics, and information evaluations) and the subjective assessments are presented in Table 2.

**Table 2:** Mobile Apps Rating Scale assessment of the included oncology mobile apps: dimension scores and overall quality.

Apps	A. Engagement (SD)	B. Functionality (SD)	C. Aesthetics (SD)	D. Information (SD)	App subjective quality (SD)	App quality, mean (SD) (A+B+C+D)/4
ONCOassist	4.10 (0.42)	4.87 (0.18)	3.66 (0.47)	4.37 (0.05)	3.75 (0.35)	4.25 (0.26)
Oncology Board Review	3.47 (0.31)	4.33 (0.38)	4.11 (0.51)	4.24 (0.41)	3.17 (0.76)	4.03 (0.39)
Hematology & Oncology Consult	3.07 (0.83)	4.50 (0.43)	4.22 (0.69)	4.12 (0.40)	2.92 (0.58)	3.98 (0.58)
Radiation Oncology Exam Review	3.73 (1.10)	4.17 (0.29)	3.55 (1.02)	4.25 (0.69)	3.08 (0.95)	3.92 (0.75)
NCCN Guidelines	3.20 (0)	3.62 (0.18)	3.83 (0.24)	4.75 (0.12)	3.87 (0.53)	3.85 (0.07)
ESMO Interactive Guidelines	3.60 (0.56)	4.62 (0.18)	3 (0.47)	4.10 (0.42)	3.87 (0.18)	3.83 (0.04)
Current Medical Diagnosis and Treatment	3.50 (0.14)	4.12 (0.53)	2.83 (0.71)	4.13 (0.38)	3.12 (0.88)	3.65 (0.37)
TNM Cancer Staging Manual	2.93 (0.12)	4.25 (0.25)	3.22 (1.02)	4.17 (0.60)	3.08 (1.01)	3.64 (0.33)
Pediatric Disease & Treatment	2.60 (0.57)	4.25 (0.35)	4.17 (0.24)	3.39 (0.15)	3.12 (0.88)	3.60 (0.25)
Radiation Oncology Q&A Review	2.93 (0.42)	4.17 (0.29)	3.67 (0.88)	3.59 (0.54)	2.66 (0.52)	3.59 (0.31)
Williams Manual of Hematology	3.20 (0.28)	4.37 (0.53)	3 (0.94)	3.58 (0.59)	2.62 (1.24)	3.53 (0.59)
All Diseases Treatments	2.50 (0.42)	4.62 (0.18)	4 (0.47)	2.95 (0.64)	2.50 (0.35)	3.52 (0.13)
Oncology Nursing Drug Handbook	3.07 (0.31)	3.75 (0.75)	3.78 (0.69)	3.11 (0.63)	2.66 (0.95)	3.43 (0.41)
Pharmacology for Nursing 2023	3.73 (0.76)	3.17 (1.42)	3.22 (1.07)	3.25 (0.66)	3.33 (1.38)	3.34 (0.91)
Washington Manual of Oncology	2.55 (0.66)	3.75 (0.54)	3 (0.82)	4.05 (0.71)	2.50 (1.43)	3.34 (0.59)
All Diseases Treatments 2023	1.90 (0.14)	4.37 (0.18)	3 (0.47)	3.20 (0.28)	2.37 (0.18)	3.12 (0.11)
Harrison’s Manual of Medicine	2.60 (0.20)	3.67 (0.38)	3 (1)	3.15 (0.53)	1.83 (0.38)	3.11 (0.27)
Fitzpatrick’s Color Atlas	2.60 (0.20)	3.33 (0.88)	2.78 (0.19)	3.12 (0.62)	2.58 (0.38)	2.96 (0.37)
Cervical Cancer Guide	2.10 (0.14)	3.50 (0)	2.67 (0.47)	3.25 (0.83)	2.13 (0.53)	2.87 (0.29)
Colon Cancer Guide	2.40 (0.28)	3.37 (0.18)	2.17 (0.24)	2.92 (0.35)	2.25 (0.71)	2.71 (0.17)
Overall mean	2.99 (0.68)	4.04 (0.65)	3.34 (0.80)	3.71 (0.69)	2.87 (0.86)	3.51 (0.54)

Notably, the scores were generally low, with an average of 3.51. Only 2 apps achieved an objective score higher than 4 (“good”), while in the subjective evaluations, none reached this threshold. The highest-rated app was ONCOassist (mean 4.25, SD 0.26). Oncology Board Review ranked second (mean 4.03, SD 0.39), followed by Hematology & Oncology Consult in third place (mean 3.98, SD 0.58). Reviewers noted that areas for potential improvement include aesthetics for ONCOassist and engagement for both Oncology Board Review and Hematology & Oncology Consult. The lowest-scoring apps were Colon Cancer Guide (2.71, SD 0.17), Cervical Cancer Guide (2.87, SD 0.29), and Fitzpatrick’s Color Atlas (mean 2.96, SD 0.37). The domain with the lowest scores for these 3 apps was engagement and subjective quality evaluation. The results are discussed below according to the different dimensions of the MARS. It is noteworthy that none of the evaluated apps attained a score of 4 in all dimensions.

### Apps’ Engagement

The apps with the highest scores in engagement were ONCOassist (mean 4.10, SD 0.42), Radiation Oncology Exam Review (mean 3.73, SD 1.10), and Pharmacology for Nursing 2023 (mean 3.73, SD 0.76), while the lowest scoring apps were All Diseases Treatments 2023 (mean 1.90, SD 0.14), Cervical Cancer Guide (mean 2.10, SD 0.14), and Colon Cancer Guide (mean 2.40, SD 0.28).

### Apps’ Functionality

The apps with the highest scores for functionality were ONCOassist (mean 4.87, SD 0.18), ESMO Interactive Guidelines (mean 4.62, SD 0.17), and All Diseases Treatments (mean 4.62, SD 0.17). Conversely, the apps with the lowest scores were Pharmacology for Nursing 2023 (mean 3.17, SD 1.42), Colon Cancer Guide (mean 3.37, SD 0.17), and Cervical Cancer Guide (mean 3.50, SD 0).

### Apps’ Aesthetics

The apps with the highest aesthetics scores were Hematology & Oncology Consult (mean 4.22, SD 0.69), Pediatric Disease & Treatment (mean 4.17, SD 0.23), and Oncology Board Review (mean 4.11, SD 0.51). The apps with the lowest scores were Colon Cancer Guide (mean 2.17, SD 0.23) and Cervical Cancer Guide (mean 2.67, SD 0.47).

### Apps’ Information

The apps with the highest scores in information were NCCN Guidelines (mean 4.75, SD 0.12), ONCOassist (mean 4.37, SD 0.05), and Radiation Oncology Exam Review (mean 4.25, SD 0.69). Conversely, the apps with the lowest scores were Colon Cancer Guide (mean 2.92, SD 0.35), All Diseases Treatments (mean 2.95, SD 0.64), and Oncology Nursing Drug Handbook (mean 3.11, SD 0.63).

### Apps Subjective Quality

The apps with the highest scores in this section are NCCN Guidelines and ESMO Interactive Guidelines (mean 3.87, SD 0.53, and SD 0.18). Next is ONCOassist (mean 3.75, SD 0.35), followed by Pharmacology for Nursing 2023 (mean 3.33, SD 1.38). In contrast, the app with the lowest subjective rating is Harrison’s Manual of Medicine (mean 1.83, SD 0.38).

## Discussion

### Principal Findings

In this study, the app review identified 221 apps between June 2023 and December 2024 following the search strategy, keywords, and parameters outlined by Cloconi et al [24]. After the screening phase, 20 apps for cancer prevention, treatment, therapy, or support for HCPs were selected in the systematic search. To explore them further, this study evaluated these apps with the MARS, a scientifically validated instrument that assesses 5 key dimensions: engagement, functionality, aesthetics, information, and subjective quality.

As a result, the ONCOassist app achieved the highest score among all the apps. Endorsed by the European Scientific Organisations such as the ESMO [28] and the European Oncology Nursing Society [29], it obtained particularly strong ratings in functionality and information. Its feature set is closely aligned with daily oncology practice, offering toxicity criteria, staging tools, prognostic calculators, and adjunctive functions that streamline clinical workflows and reduce time spent switching between resources [30]. These tools are clinically relevant, for example, in grading adverse events during treatment, estimating prognosis through staging systems, or calculating chemotherapy dosing at the point of care, which may explain their advantage over guideline-oriented, exam-oriented, or single-purpose apps. ONCOassist has also been recognized as a best practice model for incorporating user feedback in its development [30] and is valued for enhancing performance by providing guideline-based recommendations that act as a safety net for clinical decision-making [31], as well as showing potential utility in frailty assessment [32]. Its integration into oncology practice is already being discussed within professional networks [33], and its uptake is evident in some regions. As an instance, Devnani et al [34] reported that nearly 60% of a sample of 155 Indian oncology professionals had used it for over a year. Nonetheless, the available evidence should be interpreted with caution due to small samples, non-probabilistic recruitment, and potential conflicts of interest, highlighting the need for future research with more robust designs to confirm its clinical effectiveness and real-world impact.

Despite the increasing relevance of mobile apps for cancer care and management, the number of apps identified in this study is lower than that reported in previous research. Comparatively, Lima et al [4] identified 34 mobile apps published between 2015 and 2022 aimed at patients with any type of cancer for diagnosis, prognosis, monitoring, and treatment. In any case, this study focused exclusively on free apps designed for use by health professionals. In this regard, although the study by Charbonneau et al [13] identified 123 apps for cancer intended for the general public, only 3 of them are aimed at professionals. Consequently, it is to be expected that the number of apps identified would be relatively limited. Importantly, this limited number is not only consistent with prior studies but also reflects a broader structural scarcity of oncology-specific mobile apps for professionals. Much of digital oncology innovation currently takes place through web-based platforms or tools integrated into electronic health records rather than stand-alone mobile apps, which narrows the availability of dedicated mobile solutions in app stores.

The assessment of apps indicates that the quality of the identified apps is generally poor. The mean score was 3.51 (SD 0.54), with only 2 apps (the aforementioned ONCOassist app and Oncology Board Review) exceeding the 4-point threshold, which is considered to indicate a “good” level of quality. These findings align with the existing literature. Amor-García et al [35] used the same tool to evaluate 46 apps designed for patients with genitourinary tumors, obtaining a mean score of 2.98 (SD 0.77). In their study, only 13% (6 out of 46) of the apps achieved a score exceeding 4 on the MARS scale [36]. In a similar vein, Böhme et al [15] used a tool based on the MARS scale, which indicated that 60% of the apps for cancer were rated as insufficient or deficient. Similarly, the mean MARS score for the overall quality of the 88 apps addressing hematological conditions (including hematological cancers among other pathologies) in the review by Narrilos et al [37] was 3.03 (SD 1.14). They concluded that more than half of the apps do not meet acceptable criteria for quality and content, with most providing only information about the conditions and lacking interactivity and personalization features. Scholz and Teetz [14], who analyzed the quality of 33 identified mHealth apps focusing on breast cancer, agreed with the findings of previous studies as they also found problems with app quality. Additionally, results of studies that focused on the evaluation of mobile apps intended for cancer patients are similar to the results of studies that evaluated the quality of apps intended for HCPs [13]. This consistency in lack of quality in apps was also observed in this study, as none of the evaluated apps achieved a score of at least 4 across all dimensions of the MARS scale. The assurance of high-quality apps (eg, through the provision of user-friendly interfaces, the presentation of evidence to support them, or the involvement of users in their creation) would serve as a facilitator for the implementation of mHealth [38].

Beyond individual app performance, our findings also reveal differences across categories. Apps classified under prevention tended to focus on awareness and lifestyle recommendations, but they generally scored lower on functionality, reflecting a lack of interactive or decision-support features. In contrast, treatment-related apps offered more sophisticated functionalities such as toxicity grading, staging tools, or guideline-based recommendations, which explains their higher ratings in functionality and information. Finally, support apps showed strengths in accessibility and patient-oriented resources but often lacked the depth of evidence-based content required for professional use. Taken together, these patterns suggest that while treatment-oriented apps currently provide the most comprehensive toolsets for oncology professionals, prevention and support apps remain underdeveloped for clinical practice and represent important areas for future improvement. However, these comparative insights should be interpreted with caution, since the small number of apps included (n=20) restricts both the representativeness and the statistical power of the findings, as the limited sample constrains subgroup comparisons and increases the influence of outliers, making the results more descriptive than inferential. Taken together, these category patterns highlight the inherently heterogeneous nature of the digital oncology landscape. This heterogeneity can be understood as a structural feature of current app ecosystems, where tools differ sharply in purpose, evidence base, and alignment with clinical workflows.

This study is not without limitations. First, there may be additional apps not captured in this review. The keywords used for the review might not identify other relevant apps for cancer HCPs. Furthermore, while Google Play and the Apple Store account for approximately two-thirds of the mobile apps downloaded annually [39], there are other, less commonly used download platforms that were not included in this review. In addition, the review focused exclusively on free apps. While this approach reflects real-world usage patterns and aligns with the scope of the TRANSiTION project [16,17], it may also reduce the representativeness of the findings, since paid apps, which could differ in quality or functionality, were excluded. Limiting the search to English-language apps also excludes numerous potentially relevant apps in other languages, which may affect the generalizability of our findings. In particular, this restriction could underrepresent apps developed for and widely used within non–English-speaking European health care systems. However, this choice was consistent with the primary aim of the study, which is embedded in the TRANSiTION project, where the apps may be used as common resources for participants from 14 European countries. English was selected as the working language of the project and provided a common ground to ensure accessibility, comparability, and feasibility of evaluation across countries. Moreover, the number of apps evaluated after the screening process (n=20) is relatively small. This limits the representativeness of the sample and reduces the statistical power of the findings, as subgroup analyses could not be conducted, and outliers may disproportionately affect the results. Therefore, the study should be viewed primarily as descriptive. Future research with larger and more diverse samples, ideally through systematic surveillance and international collaborations, is needed to provide a more comprehensive and robust overview of oncology apps. Second, although the evaluation of the apps was conducted using a standardized procedure and a validated tool, the evaluators may be susceptible to response biases, as the assessment relies on subjective ratings. In addition, the MARS assesses the app’s evidence base with a single item (item 19), which appears insufficient for determining whether an app should be included in an HCP’s resource portfolio. These limitations imply that the validity and reliability of the quality scores may be constrained, since rater bias and the restricted appraisal of evidence can influence results. Consequently, MARS ratings should be understood as standardized indicators of app quality rather than definitive evidence of clinical effectiveness or robustness. Nevertheless, MARS is the most widely used among all app evaluation scales and is suitable for general public use [40]. Future research should therefore combine standardized quality assessments with empirical studies to determine the actual impact of oncology apps on professional performance, efficiency, and well-being.

Health apps are emerging technologies that handle sensitive personal health data, which can significantly impact health outcomes and are susceptible to cybersecurity threats [41]. This study highlights that while some apps have limited supporting evidence from previous studies, none have provided evidence of their validity and reliability for their intended purpose. In the study of Berger-Groch et al [42] that focused on identifying mobile apps that can be used in the treatment of patients with musculoskeletal tumors, only 3 scientific articles on mobile apps in orthopedic oncology were present, yet several more apps were available without scientific medical evaluation. Some of the justifications provided in the literature are that apps tend to become unavailable in app stores once the studies have concluded, or that a significant proportion of the papers do not reveal the specific apps that were used [11]. The lack of regulatory standards or frameworks for assessing the quality of these apps is, therefore, of serious concern [43] and has been described as the “Wild West of healthcare” [44]. There are some initiatives to regulate the quality of apps, such as those in the National Health Service [45], the Spanish Ministry of Health and Agència de Qualitat i Avaluació Sanitàries de Catalunya [46], or the ongoing European project ASSESS DHT [47], which focuses precisely on filling this gap.

In addition to these regulatory challenges, the predominance of commercially developed apps in our sample (18 out of 20) raises concerns regarding both content quality and sustainability. On the one hand, commercial investment may provide resources for more advanced design and maintenance. On the other hand, it can introduce potential conflicts of interest, with content that may prioritize promotional goals over evidence-based practice. Sustainability is also uncertain, as the long-term availability of commercially driven apps depends on market viability, a factor that may explain why many oncology apps become unavailable after only a few years. Similarly, apps developed within the framework of publicly funded research projects may encounter comparable sustainability challenges. Although grounded in scientific evidence, their maintenance and updates are often limited to the project’s duration, after which support may be discontinued, restricting their long-term utility. Prior research has shown that such discontinuity contributes to poor adoption rates and a lack of long-term sustainability, highlighting the need for evidence-informed methodological approaches that extend beyond funding cycles to ensure continuity and adoption in real-world practice [48]. These findings underscore the importance of regulatory oversight and sustainable development models to guarantee that oncology professionals can rely on digital tools with transparent, evidence-based, and durable content. Overall, the scarcity and heterogeneity identified in this review underscore the fragmented nature of the current digital oncology ecosystem and highlight the need for coordinated development and robust evaluation frameworks.

From a clinical perspective, our findings highlight the need for oncology professionals to exercise caution when selecting mobile apps, giving priority to those that demonstrate transparent evidence and functionalities that support clinical workflows. In this sense, the implementation of regulatory frameworks and validated quality standards will be crucial for transforming our results into practical guidance, ensuring that HCPs can safely rely on digital tools that enhance efficiency and patient care. In this context, this study, as part of the TRANSiTION project, which incorporates multiprofessional and patient perspectives from several European Union Member States into its design and implementation, as well as in the dissemination and communication of its outcomes, will contribute to initiating new research lines and developing apps with enhanced methodologies [49].

### Conclusions

This study identified mobile apps available to cancer care HCPs and highlighted their limitations. Currently, there is insufficient evidence in the literature to support the effectiveness of any specific downloadable app. However, this review offers a structured evaluation of app quality and a comparative overview of each app’s strengths and limitations, providing a baseline for future research and development in this field. While overall app quality was poor, the findings point to the potential usefulness of the ONCOassist app, which emerged as the highest-rated app in our assessment and merits further investigation through robust empirical studies. Continued monitoring, evidence-informed development, and the implementation of quality standards will be essential to ensure that mobile apps can reliably support oncology practice in the future.

## Data Availability

Data supporting the results reported in the article are available on request from the authors.

## Appendix

Multimedia Appendix 1 PRISMA checklist.

Multimedia Appendix 2 Excluded apps.

## Abbreviations

ESMO: European Society for Medical Oncology
HCP: health care professional
ICC: intraclass correlation coefficient
MARS: Mobile Apps Rating Scale
mHealth: mobile health
PRISMA: Preferred Reporting Items for Systematic Reviews and Meta-Analyses
TRANSiTION: Digital Transition and Digital resilience in Oncology
