# Supplementary material for: Mobile Apps for Oncology Health Care Professionals: Mapping and Assessment Study
Source: JMIR Mhealth Uhealth. 2026 Feb 11;14:e71203. doi: 10.2196/71203 (PMC12936660; doi:10.2196/71203)
Supplement: Multimedia Appendix 2 [file mhealth_v14i1e71203_app2.docx]

**Table S1.**

| **App** | **Platform** | **Target Group** | **Created** | **Updated** | **Reason for exclusion** |
| --- | --- | --- | --- | --- | --- |
| ***Cancer Nursing 2000 Flashcards*** | Android | Nurses | NA | Jan, 2019 | Not available |
| ***Cancer & Oncology Nursing*** | Android | Cancer & Oncology Nurses | 2018 | May, 2023 | Not free of charge |
| ***Cancer & Oncology Nursing Exam*** | Android | Cancer & Oncology Nurses | 2018 | Sep, 2018 | Not available |
| ***Cancer Therapy Advisor*** | Android | Oncology professionals | 2012 | Aug, 2021 | Not available |
| ***Go-Exap app*** | Android & iOS | HCPs | 2021 | May, 2023 | It requires administrator privileges to use |
| ***Manual of Clinical Oncology*** | Android | HCPs | 2014 | Jan, 2019 | Not free of charge |
| ***NBIA Data Retriever app*** | iOS | Researchers/ Health care professionals | 2018 | Oct, 2022 | Technical issues prevented it from being used |
| ***Nursing Management in Cancer Care*** | Android | Nurses | NA | Jan, 2019 | Not available |
| ***Nursing Management In Cancer Care PremIum*** | Android | Nurses | NA | Jan, 2019 | Not free of charge |
